# A Unique Enhancement of *Propionibacterium freudenreichii*’s Ability to Remove Pb(II) from Aqueous Solution by Tween 80 Treatment

**DOI:** 10.3390/ijms23169207

**Published:** 2022-08-16

**Authors:** Fanny George, Marie Titécat, Nicolas Barois, Catherine Daniel, Anne Garat, Gwénaël Jan, Benoît Foligné

**Affiliations:** 1 Univ. Lille, Inserm, CHU Lille, U1286-INFINITE-Institute for Translational Research in Inflammation, F-59000 Lille, France; 2 Univ. Lille, CNRS, Inserm, CHU Lille, Institut Pasteur de Lille, U1019-UMR 9017-Center for Infection and Immunity of Lille, F-59000 Lille, France; 3 Univ. Lille, CHU Lille, Institut Pasteur de Lille, ULR 4483-IMPECS-IMPact de l’Environnement Chimique sur la Santé Humaine, F-59000 Lille, France; 4 CHU Lille, Unité Fonctionnelle de Toxicologie, F-59000 Lille, France; 5 STLO, INRAE, Agrocampus Ouest, Institut Agro, Science & Technologie du Lait & de l’Œuf, F-35000 Rennes, France

**Keywords:** bioremediation, lead removal, *Propionibacterium freudenreichii*, polysorbate 80, Tween 80

## Abstract

Microbial agents have promise for the bioremediation of Pb(II)-polluted environments and wastewater, the biodecontamination of foods, and the alleviation of toxicity in living organisms. The dairy bacterium *Propionibacterium freudenreichii* is poorly able to remove Pb(II) from aqueous solution at 25 ppm, ranging from 0 to 10% of initial concentration. Here, we report on an original strong enhancement of this activity (ranging from 75% to 93%, *p* < 0.01) following the addition of a polysorbate detergent (Tween^®^ 80) during or either shortly after the growth of a *P. freudenreichii* culture. We evaluated the optimal Tween^®^ 80 concentration for pretreatment conditions, documented the role of other detergents, and explored the possible mechanisms involved. Our results reveal a novel, environmentally friendly, low-cost pretreatment procedure for enhancing the selective removal of lead from water by probiotic-documented bacteria.

## 1. Introduction

The widespread heavy metal lead (Pb) is regarded as “probably carcinogenic” by the United States Environmental Protection Agency and has been classified as a group 2A substance by the International Agency for Research on Cancer. Acute inorganic Pb poisoning and Pb contamination as a metallic trace element are currently public health and environmental concerns in both industrialized and developing countries. Safe drinking water should contain less than 10 µg·L^−1^ Pb—a threshold that is very often exceeded [1]. In fact, there is no “safe” level of exposure to Pb. Chronic Pb exposure results in toxic effects on the nervous system (including neuropsychiatric disorders and mental retardation in children), anemia, high blood pressure, persistent vomiting, infertility, and liver and kidney disorders [2,3,4]. It was recently shown that ingested Pb also exerts subtle, harmful effects through inflammation, oxidative stress, impacting gut physiology and the microbiome [5,6]; in turn, this leads to dysbiosis and heightened susceptibility to many chronic diseases.

In order to lower the bioavailability and toxicity of Pb in general and Pb(II) in particular, the metal can be removed from wastewater and contaminated matrices by applying physicochemical methods such as membrane filtration, ion exchange, coagulation, adsorption, and precipitation [7]. These remediation techniques are limited by their low efficiency and high operating costs. Furthermore, the non-regenerable materials used in these processes are not environmentally friendly. Various adsorbents (including carbon nanotubes, minerals, and biomolecules) have also been widely used to remove Pb(II), and also exhibited distinct performances.

Compared with these conventional methods, the use of bacterial biosorbents for heavy metals is innovative and cost-effective [7,8,9]. Several passive and active mechanisms (such as adsorption and bioaccumulation) are involved in these bacteria-based methods. Whereas the use of environmentally sourced bacteria has been explored for wastewater remediation, food-grade microbes are mandatory (or at least strongly recommended) for probiotic-like “within the gut” bioremediation [10,11,12]. In this context, extensive studies of lactic acid bacteria and bifidobacteria have revealed the abilities of specific strains to remove heavy metal ions from aqueous solutions. This is consistent with the huge diversity of intrinsic surface molecules capable of binding anions (e.g., polysaccharides and proteins) and metal accumulation processes (e.g., active ion uptake and intracellular ion sequestration via substances like polyphosphates) [11,13,14,15,16]. It is noteworthy that bacterial adsorption processes are also influenced by many factors, including the pH, temperature, salt concentration, osmolarity, and bacteria–metal contact time [17,18]. Depending on which specific environmental conditions are involved (i.e., wastewater, a food matrix, or the intestinal niche), bacteria-based Pb removal can be modulated and/or optimized for a given bacterial strain. For example, Pb(II) removal from aqueous solution by a strain of *Pseudomonas aeruginosa* was enhanced by up to 40% following supplementation of the growth medium with K_2_SO_4_ [19]. This enhancement was due to changes in the functional groups on the bacterial surface.

The dairy bacterium *Propionibacterium freudenreichii* is a Gram-positive, non-spore-forming, anaerobic-to-aerotolerant member of the actinomycetales. It is used as a cheese ripening starter and, on the basis of a long history of safe use in food, has gained “generally recognized as safe” (GRAS) status based on the long history of safe utilization in food. *Propionibacterium freudenreichii* has also been given “qualified presumption of safety” (QPS) status by the European Food Safety Authority. Overall, *P. freudenreichii* has attracted recent attention as a probiotic with demonstrated antimicrobial and anti-inflammatory properties, the secretion of bifidogenic prebiotic metabolites, and a potential tool for toxin bioremediation [20,21,22,23]. It has also been reported that one strain of *P. freudenreichii* can (alone or when combined with lactobacilli) remove heavy metals (including Pb) from a matrix [24,25]. However, we recently screened more than 200 strains of lactic acid bacteria, bifidobacteria, and propionibacteria for their ability to potentially bioremediate Pb, cadmium (Cd), and aluminum (Al); none of the 20 *P. freudenreichii* strains removed 25 ppm Cd, Al, or Pb effectively. In contrast, some *Lactobacillus* strains were strong Pb chelators and removed up to 80% of the metal from a 25 ppm solution whereas other *Lactobacillus* strains could not remove more than 10% of the initial amount of metal [26].

Tween^®^ 80 (frequently referred to as polysorbate 80, polyoxyethylene (20) sorbitan monooleate, or polyethylene glycol sorbitan monooleate) is a non-ionic surfactant and emulsifier used as a food additive (EU number E433). Tween^®^ 80 is widely used in microbiology (mostly at a concentration of 0.1%) to increase the growth rate of bacteria (such as *Mycobacterium tuberculosis* or *Lactobacillus* ssp.) when added to the culture medium; the detergent serves as an alternative energy source via the provision of exogenous oleic acid. For example, almost all de Man Rogosa Sharpe (MRS) media (the main commercial culture medium currently used to grow lactobacilli and bifidobacterial) contain 0.1% Tween^®^ 80. Furthermore, Tween^®^ 80 is used empirically to reduce bacterial cell aggregation and to protect against stress [27,28]. At concentrations of up to 0.5%, Tween^®^ 80 can exert bactericidal activity and decreases the minimal bactericidal concentration of a range of antibiotics with activity against staphylococci and *Helicobacter pylori* strains [29,30]. Lastly, it was recently reported that the addition of Tween^®^ 80 to the culture medium enhanced anthracene biodegradation by *Sphingomonas* sp. [31,32,33]. However, the physiological and/or biophysical mechanisms underlying most of Tween^®^ 80’s above-mentioned effects have not been fully elucidated.

Here, we report on a strong enhancement of *P. freudenreichii*’s ability to remove Pb(II) from aqueous solution via culture medium supplementation with low Tween^®^ 80 concentrations. The objectives of the present study were: (i) to identify the optimal Tween^®^ 80 concentration and conditions; (ii) to compare Tween^®^ 80’s effects on metal bioremediation with those of other detergents; and (iii) to consider possible mechanisms of Pb removal by *P. freudenreichii* and other bacteria (i.e., lactobacilli, bifidobacteria, cutibacteria, and enterobacterales) in the presence or absence of Tween^®^ 80.

## 2. Results

### 2.1. Tween^®^ 80 Markedly Enhances P. freudenreichii’s Pb(II) Removal

**Figure 1:**
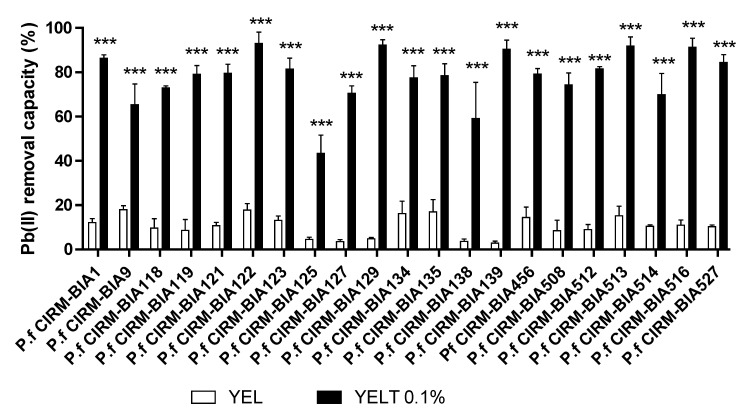
The Pb(II) removal ability of 21 *P. freudenreichii* strains grown in YEL medium in the absence of Tween^®^ 80 (white bars) or in YEL medium containing 0.1% Tween^®^ 80 (YELT 0.1%; black bars). Data are expressed as the mean ± SD % of Pb^2+^ removed from the solution (starting concentration: 25 ppm) in triplicate experiments (see the Methods section). *** *p* < 0.001 for YEL vs. YELT 0.1% with the indicated strain.

The Pb(II) removal ability of 21 *P. freudenreichii* strains cultured in YEL medium alone was relatively weak, with a mean ± SD (range) removal value of 10.3% ± 6.5 (2%–18%) according to inter-strain differences (Figure 1). In contrast, all strains demonstrated very markedly greater Pb removal abilities when cultured with YEL + 0.1% Tween^®^ 80 (YELT 0.1%); the removal ability was around 80% on average and ranged from 43% to 93%. For individual strains, Tween^®^ 80 enhanced Pb removal ability by a factor of 6 to 25 (*p* < 0.001) (Figure 1). It is noteworthy that the bacteria were washed thoroughly (5 times) before the removal assays, and so residual compounds from the bacterial broth had been removed. Hence, only Tween^®^ 80 could have been responsible for markedly enhancing the strains’ removal ability and reducing inter-strain differences.

### 2.2. Tween^®^ 80 Prior Pb(II) Exposure Is Sufficient to Enhance P. freudenreichii’s Pb(II) Removal 

**Figure 2:**
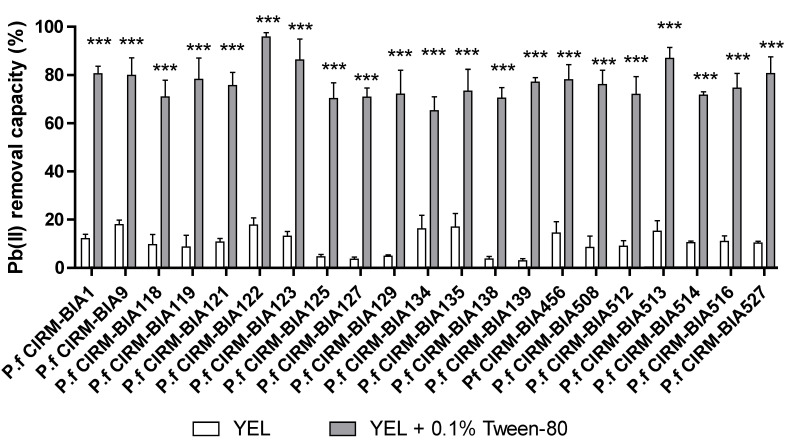
The Pb(II) removal ability of 21 *P. freudenreichii* strains treated with Tween^®^ 80. Bacteria were grown in YEL medium (white bars) alone and then treated (or not) with 0.1% Tween^®^ 80 (YEL + 0.1%) (grey bars). Data are expressed as the mean ± SD % of Pb^2+^ removed from the solution (starting concentration: 25 ppm) in triplicate experiments (see the Methods section). *** *p* < 0.001 for YEL vs. YEL + 0.1%T, with the indicated strain.

We also evaluated the 21 *P. freudenreichii* strains’ ability to remove Pb(II) salts when Tween^®^ 80 was added immediately before exposure to the lead solution (giving a final concentration of 0.1% (YEL + Tween 0.1%). Bacteria harvested during the early stationary phase and then mixed with Tween^®^ 80 demonstrated an enhanced ability to remove Pb, relative to control experiments in the absence of Tween^®^ 80 (Figure 2).

### 2.3. Influence of the Tween^®^ 80 Concentration, the Pb Concentration, and the Bacteria-Tween^®^ 80 Contact Time

**Figure 3:**
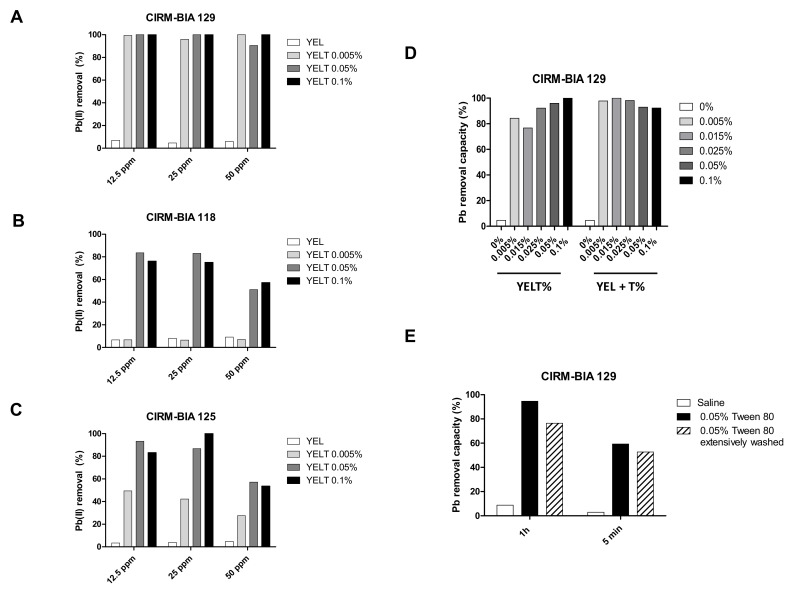
The Pb(II) removal ability of three *P. freudenreichii* strains as a function of the lead concentration, Tween^®^ 80 concentration, and contact time. (**A**) strain CIRM-BIA 129, (**B**) strain CIRM-BIA 118, and (**C**) strain CIRM-BIA 125 were grown in YEL alone or in YEL medium containing 0.1% Tween^®^ 80 (YELT), prior to exposure to 12.5, 25, and 50 ppm Pb(II). (**D**) Comparison of various concentrations of Tween^®^ 80 added to the medium during culture or added at the same time as the metal ion solution, for *P. freudenreichii* CIRM-BIA 129. (**E**) Impact of the bacteria–0.05% Tween^®^ 80 contact time (1 h or 5 min) and extensive washing on CIRM-BIA 129’s ability to remove Pb^2+^. Representative data are expressed as the % of Pb^2+^ removed from the solution.

An enhancement was also observed after exposure to various Pb concentrations (12.5 ppm, 25 ppm and 50 ppm) and with various (and lower) Tween^®^ 80 concentrations (0.005%, 0.05%, and 0.1%) in the culture medium for three selected *P. freudenreichii* strains (CIRM-BIA 129, CIRM-BIA 118, and CIRM-BIA 125). For *P. freudenreichii* CIRM-BIA 129, the enhancement was similar at 0.005%, 0.05% and 0.1% Tween^®^ 80, and almost 100% of the Pb(II) was removed at all concentrations tested (Figure 3A). For CIRM-BIA 118, the lowest concentration of Tween^®^ 80 (0.005%) was not sufficient for Pb removal; a concentration of at least 0.05% detergent was required for the best removal ability at 12.5, 25, and (to a lesser extent) 50 ppm (Figure 3B). Considering the CIRM-BIA 125 strain, 0.005% Tween^®^ 80 induced only partial removal of Pb at 12.5 and 25 ppm (Figure 3C), whereas 0.05% and 0.1% Tween^®^ 80 fully enhanced the maximal metal removal ability. In contrast to CIRM-BIA 129, 0.05% and even 0.1% Tween^®^ 80 failed to induce the total removal of 50 ppm Pb(II) by CIRM-BIA 125—suggesting that the Pb concentration is also a limiting factor in the chelation process. Overall, our results suggest that Tween^®^ 80’s effect on Pb removal by *P. freudenreichii* is somewhat dependent on both the detergent concentration and the metal concentration of the metal, in a manner that differs from one strain to another. Given the CIRM-BIA 129 strain’s ability to remove Pb(II) at the lowest dose (0.005%) of Tween^®^ 80, we selected it for further experiments. It is noteworthy that the effect of the lowest dose of Tween^®^ 80 in the culture broth was greater when the detergent was added an hour before the metal assay (as shown for strain CIRM-BIA 129 in Figure 3D). The bacteria–Tween^®^ 80 contact time clearly influenced the efficiency of Pb(II) removal; the effect was lower after 5 min of treatment than after an hour (Figure 3E). Interestingly, these effects were similar when bacteria were extensively washed (at least 6 times) with saline buffer to remove the detergent—suggesting that exposure to Tween^®^ 80 alters the bacteria’s Pb(II) removal ability in a lasting manner (Figure 3E).

### 2.4. Tween^®^ 80 Has No Impact on Other Metals Removal by P. freudenreichii’s

**Figure 4:**
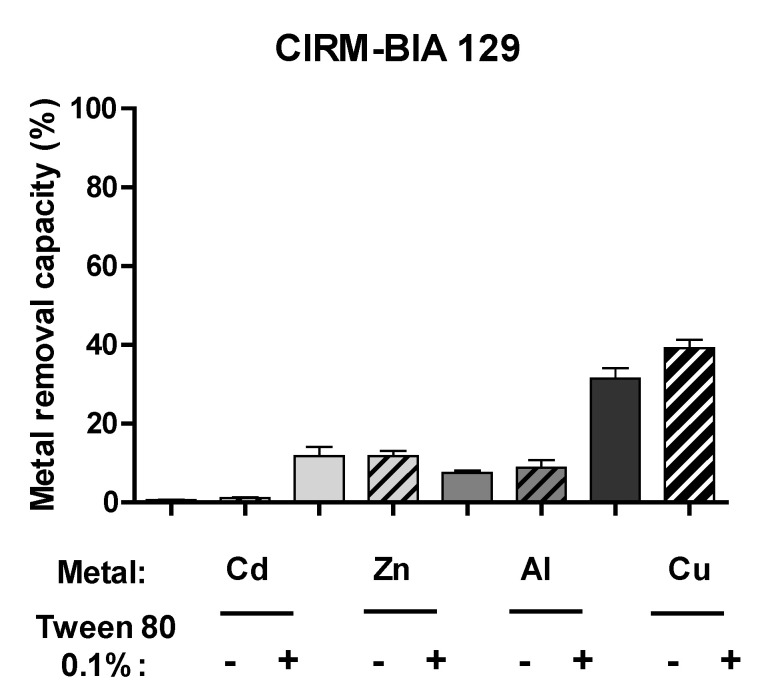
*P. freudenreichii* CIRM-BIA 129’s ability to remove Cd(II), Zn(II) Al(III) and Cu(II) with (hatched bars) our without (solid bars) 0.1% Tween^®^ 80 treatment. The data are expressed as the mean ± SD % of metal removed from the solution (starting concentration: 25 ppm) in triplicate experiments.

Using the *P. freudenreichii* strain CIRM-BIA 129, we evaluated the impact of 0.1% Tween^®^ 80 on the ability to remove other divalent cation metals: Cd(II), Cu (II), Zn(II), and aluminum Al(III). As shown in Figure 4, the presence of Tween^®^ 80 had a weak effect for all four metal ions tested. In contrast to lead, removal of cadmium was still very low in both the presence and absence of Tween^®^ 80. This was also the case for several other *P. freudenreichii* strains (data not shown), which highly suggests that the Tween^®^ 80-mediated enhancement phenomenon is highly specific for Pb(II).

### 2.5. Tween^®^ 80 Impact on Pb(II) Removal Is Bacterium Specific

**Figure 5:**
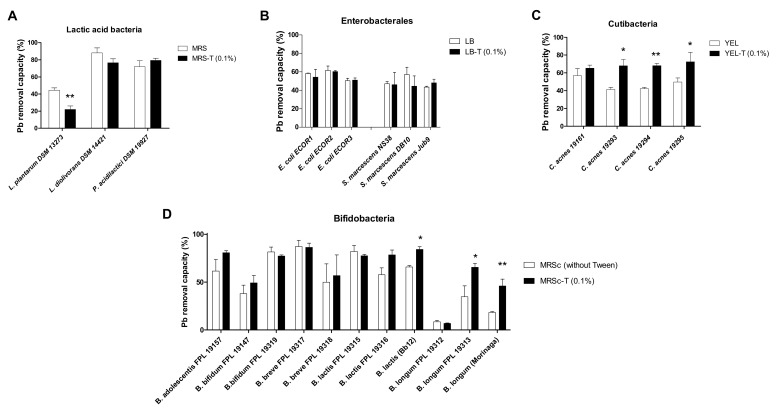
The Pb(II) removal ability of various bacterial strains grown with Tween^®^ 80. (**A**) lactic acid bacteria were grown in MRS medium (white bars) or in MRS medium containing 0.1% Tween^®^ 80 (MRS-T 0.1%, black bars). (**B**) Enterobacterales (i.e., *E. coli* and *S. marcescens* strains) were grown in LB medium (white bars) or in LB containing 0.1% Tween^®^ 80 (LB-T 0.1%, black bars). (**C**) *Cutibacterium acnes* strains were grown in YEL medium (white bars) or in YEL containing 0.1% Tween^®^ 80 (YEL-T 0.1%, black bars). (**D**) Eleven bifidobacteria strains from five species were grown in MRSc medium (white bars) or in MRS medium containing 0.1% Tween^®^ 80 (MRSc-T 0.1%, black bars). The data are expressed as the mean ± SD % of metal removed from the solution (starting concentration: 25 ppm) in triplicate experiments. * *p* < 0.05; ** *p* < 0.01 for Tween-free media vs. the same media with 0.1% Tween^®^ 80, in each strain.

We next addressed the possible impact of Tween^®^ 80 on other bacteria’s ability to remove Pb(II). Since 0.1% Tween^®^ 80 is present in nearly all the MRS media used to cultivate lactic acid bacteria and bifidobacteria on a commercial basis, we selected this medium free of Tween^®^ 80 for our experiments. Surprisingly, supplementation of MRS medium with 0.1% Tween^®^ 80 did not have a marked influence on Pb(II) removal by the two *Lactobacillus* and the *Pediococcus* strains tested; in fact, it even decreased Pb(II) removal by *L. plantarum* DSM 13273 by a factor of 2 (*p* < 0.01) (Figure 5A). Similarly, the addition of Tween^®^ 80 to the culture medium of Gram-negative *Enterobacterales* (i.e., *E. coli* and *S. marcescens* strains) did not modify the bacteria’s intrinsic ability to remove Pb ions from aqueous solution (Figure 5B). We next considered several strains of *Cutibacterium acnes* (formerly known as *Propionibacterium acnes*); in taxonomic terms, these are very similar to the dairy *P. freudenreichii* because all are members of the order *Propionibacteriales* and the family *Propionibacteriaceae*. The presence of Tween^®^ 80 had a null or weak effect on metal removal, with 1.1- to 1.6-fold enhancements (Figure 5C). The bifidobacteria belong to the same phylum (Actinobacteria) as the Propionibacteria; we therefore tested a set of 11 bifidobacterial strains (from four distinct species). With the exception of slight but significant enhancements in two *B. longum* strains (1.8- and 2.5-fold; *p* < 0.01), the presence of 0.1% Tween^®^ 80 did not change the Pb(II) removal ability in strains with a low or high level of activity at baseline (Figure 5D). It is noteworthy that the results were similar when Tween^®^ 80 was added extemporaneously, at the same time as the metal solution (i.e., no pre-incubation with the bacteria; data not shown).

### 2.6. Other Detergents Differ in Their Effects on Pb(II) Removal by P. freudenreichii

**Figure 6:**
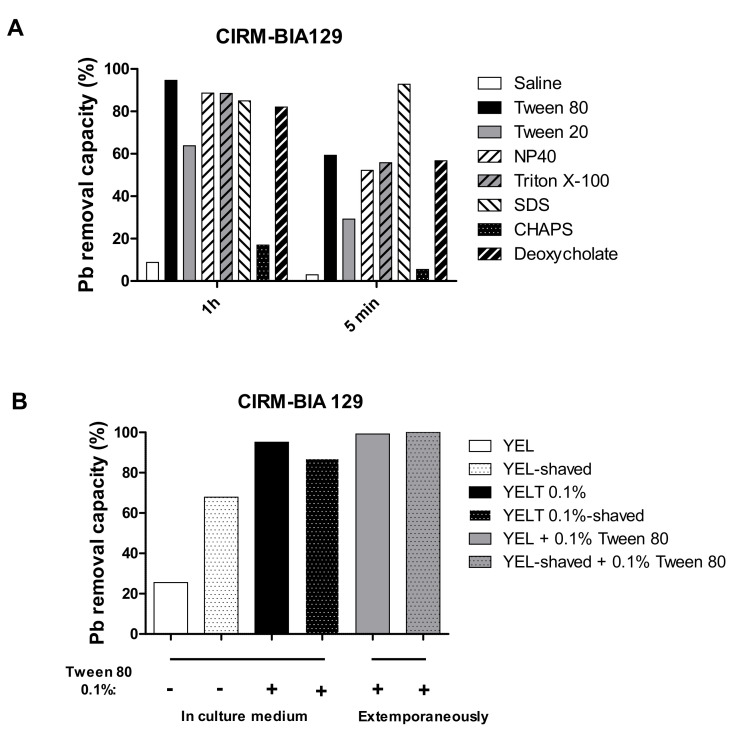
*P. freudenreichii*’s Pb(II) removal ability varies as a function of the detergent and the surface protein treatment. (**A**) The effects of seven different detergents (0.05%, added extemporaneously) for 1 h or for 5 min) are shown for the *P. freudenreichii* strain CIRM-BIA 129. (**B**) CIRM-BIA 129 grown in YEL (white bar) and then “shaved” with trypsin (dotted white bar), grown in YEL, pre-incubated with Tween^®^ 80 (black bar), and then shaved (dotted black bar), or grown in YEL, exposed to 0.1% Tween^®^ 80 and the metal ion solution at the same time (grey bar), and then shaved (dotted grey bars). Representative data are expressed as the % of Pb^2+^ removed from the 25 ppm initial solution.

As Pb(II) removal was greatest for the post-culture exposure to 0.05% Tween^®^ 80 and a bacteria-Tween^®^ 80 contact time of 1 h, we compared several detergents under the same conditions (i.e., exposure to 0.05% Tween^®^ 80 for 5 min or 1 h; Figure 6A). Tween^®^ 20 was less efficient than Tween^®^ 80 at both contact times. In contrast, the two nonionic detergents Nonidet-P40 and Triton X-100 and the anionic bile salt deoxycholate gave much the same overall enhancement of Pb(II) removal ability as Tween^®^ 80. The anionic sodium dodecyl sulfate (SDS) appeared to be even more effective because it led to a maximal effect after a 5-min contact time. Lastly, the zwitterionic detergent 3-((3-cholamidopropyl) dimethylammonio)-1-propanesulfonate (CHAPS) did not have a notable impact on metal removal.

### 2.7. Contribution of Cell Surface Integrity to the Pb(II) Removal Mechanism

To test the involvement of surface proteins in Pb(II) removal, the *P. freudenreichii* strain CIRM-BIA 129 was subjected (or not) to trypsin-catalyzed shaving before the metal removal assays. Mild trypsin digestion alone was associated with greater levels of Pb removal (up to 3-fold), suggesting that exposed surface proteins reduce cell wall-metal interactions under non-shaved conditions (Figure 6B). The presence of 0.1% Tween^®^ 80 was associated with a relative increase in metal removal ability, both when it was present in the culture medium before the shaving step and when it was added during the shaving step (Figure 6B). Hence, our results show that the detergent’s influence on metal immobilization by *P. freudenreichii* did not depend on the bacterial surface proteins. The zeta potential (ζ) is widely used as a proxy parameter for bacterial cell surface charge. Nonspecific adsorption of ions or polyelectrolytes onto the cell surface, however, alters the value and polarity of the measured zeta potential, according to electrophoretic mobility. Here, we explore the possible modification of the intrinsic charge by the detergent.

In order to determine whether the net surface charge was altered by the presence of Tween^®^ 80, we performed zeta-potential (ζ) assays under various conditions. The CIRM-BIA 129 strain exhibited a zeta potential of −8.26 ± 0.57 mV in pH 7 saline buffer and −9.06 ± 1.36 mV at pH 8, i.e., a negative net charge at baseline. Treatment with 0.1% Tween^®^ 80 significantly altered the zeta potential, which was −10.47 ± 0.73 mV at pH 7 (*p* < 0.05) and −10.68 ± 1.61 mV at pH 8 (*p* < 0.05); the electronegativity at neutral pH was nearly 25% greater. It is noteworthy that “shaved” bacteria were less electronegative, with values of −7.54 ± 0.47 mV at pH 7 (*p* < 0.05) and −7.85 ± 0.42 mV at pH 8 (*p* < 0.05); this is in line with disorganization of the dominant S-layer and other proteins at the bacterial cell surface.

### 2.8. Morphological Appearance of Bacteria Exposed to Tween^®^ 80

**Figure 7:**
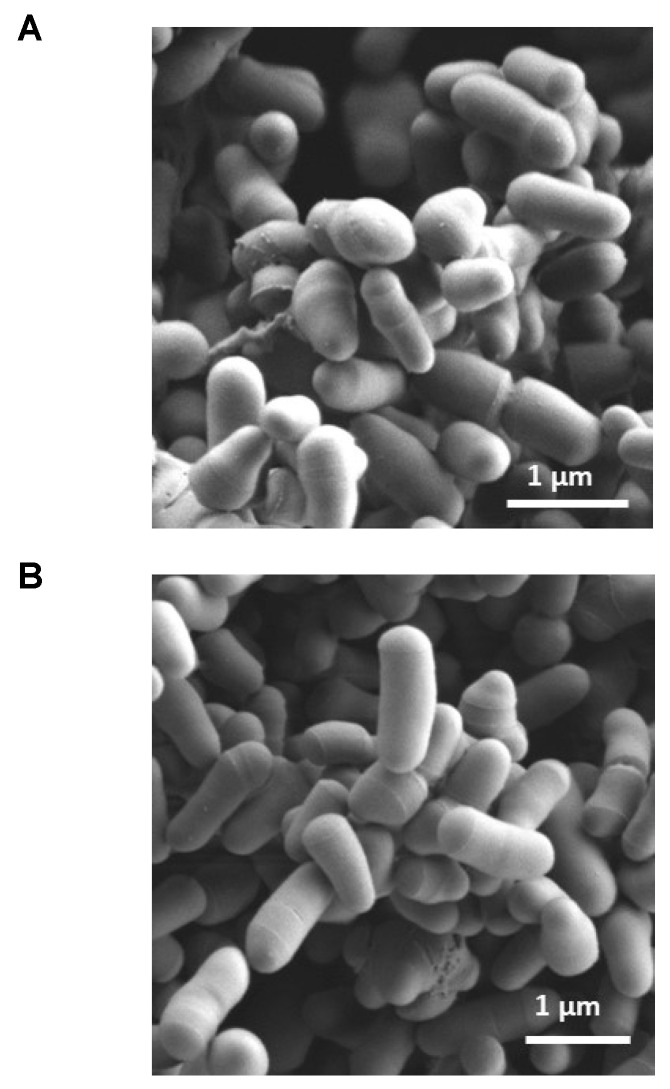
Scanning electron micrographs of *P. freudenreichii* CIRM-BIA 129. (**A**) Control bacteria. (**B**) Bacteria pretreated with 0.1% Tween^®^ 80 and then exposed to 25 ppm Pb(II). The scale bar corresponds to 1 µm.

Exposure of *P. freudenreichii* CIRM-BIA 129 to Tween^®^ 80 did not modify aggregation of the bacteria (evaluated as the median time required for spontaneous sedimentation in a liquid suspension) or the macroscopic aspect and size of bacterial colonies plated on solid media (data not shown). Light microscopy after Gram staining neither reveals any particular morphological features due to treatment with Tween^®^ 80 and 25 ppm Pb(II). Accordingly, scanning electron micrographs showed the characteristic morphology of dairy propionibacteria (pleomorphic rods arranged in “Chinese ideograms”) and did not evidence any obvious changes in bacterium size or cell wall smoothness. It is noteworthy that no Pb(II) ion precipitates were visible on the outer surface of the cell, in accordance with poor adsorption of Pb(II) (Figure 7A,B).

**Figure 8:**
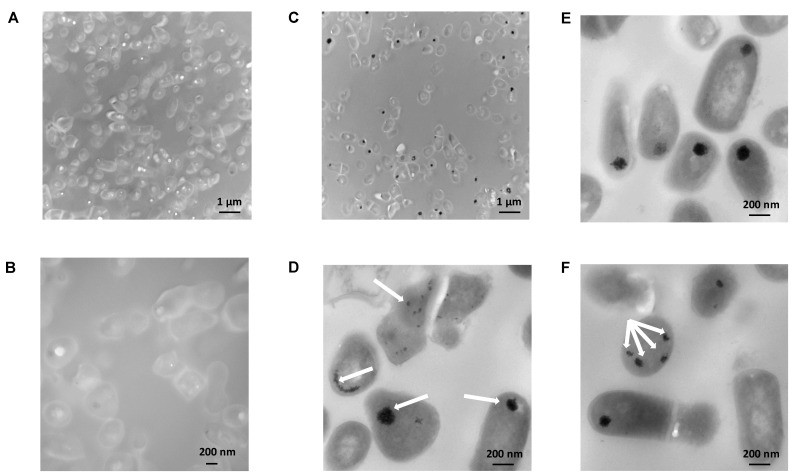
Transmission electron microscopy (TEM) observation of *P. freudenreichii* CIRM-BIA 129. (**A**) Control bacteria exposed to 25 ppm Pb(II): low magnification image. (**B**) Control bacteria exposed to 25 ppm Pb(II): high magnification image. (**C**) Bacteria treated with 0.1% Tween^®^ 80 and exposed to 25 ppm Pb(II): low magnification image. (**D**–**F**) Bacteria treated with 0.1% Tween^®^ 80 and exposed to 25 ppm Pb(II); high magnification image. The scale bar corresponds to 1 µm or 200 nm. Arrows indicate metal deposits.

Similarly, TEM did not evidence obvious metal deposits on or in propionic bacteria treated with 25 ppm of Pb(II) in the absence of detergent, regardless of the magnification (Figure 8A,B). Following treatment with Tween^®^ 80, however, TEM revealed electron-dense Pb granules mostly clustered within nearly half of the bacteria (Figure 8C,D, see arrows). Whereas a few single deposits were located near the cell wall, several agglomerates per cell were also visible (Figure 8E,F, see arrows). This observation strongly suggests that Tween^®^ 80-induced Pb removal acts preferentially by concentrating the metal in the cytoplasm, rather than promoting external binding.

## 3. Discussion

### 3.1. General Overview

We identified an innovative treatment that strongly enhanced *P. freudenreichii*’s usually poor ability to remove Pb(II) from aqueous solution. Indeed, an earlier study showed that all the screened strains of propionibacteria were poorly able to remove various heavy metal ions [26]. Here, we found that exposure to Tween^®^ 80 was associated with a strong enhancement of *P. freudenreichii*’s removal ability, with high absolute values. Strikingly, this enhancement effect was seen for lead but not for other divalent cations, such as cadmium, copper, and zinc. It is noteworthy that the effect of the Tween^®^ 80 was long-lasting, even after a short contact time; it was still present after 96 h and after the bacteria had been extensively washed. Many methods for improving the adsorption of metals by microorganisms have been described, such as the enhancement of extracellular polymeric substances in *Pseudoalteromonas* sp by the addition of sucrose to the culture medium [34]. The ability of baker yeast biomass to adsorb Cd(II) and Pb(II) was enhanced by crosslinking cystine with glutaraldehyde [35] or by treatment with ethanol and heat [36], whereas supplementation of *Pseudomonas sp*’s medium with sulfate boosted Pb(II) biosorption by increasing the number of sulfur-containing functional groups on the bacterial surface [19]. A higher percentage of peptidoglycan and teichoic acid molecules in the cell wall of Gram-positive bacteria [35] helps them bind and absorb more metal than Gram-negative species [10]. The conventional use of untreated bacteria (i.e., lactic acid bacteria and *Bacillus* sp.) for heavy metal biosorption and/or removal has been well described. However, to the best of our knowledge, there are (i) few specific treatments for enhancing the intrinsic performance of Gram-positive bacteria and (ii) no such treatments applied for probiotic-related bacteria. Moreover, the effect of Tween^®^ 80 discovered here did not correspond to the enhancement of a good underlying level of ability; in fact, exposure to Tween^®^ 80 gave *P. freundenreichii* a new property. Although 0.05% Tween^®^ 80 enhanced the activity of all *P. freundenreichii* strains, lower concentrations also had an effect in some cases. Lastly, Tween^®^ 80 enhanced the removal activity for *Bifidobacterium* spp. and the taxonomically close *Cutibacterium acnes* but not for lactic acid bacteria or Gram-negative gamma-proteobacteria.

Biosurfactants sourced from various bacteria have been shown to be useful for heavy metal bioremediation, amongst other applications [36]. However, this process cannot account for our present observations. Although some (but not all) of the *P. freudenreichii* strains tested here do produce β-D-glucan exopolysaccharides [37], Tween^®^ 80’s enhancement is extended to all strains. A similar strategy with Tween^®^ 20 was recently used to promote adsorption, uptake, and further degradation of hydrophobic polycyclic aromatic hydrocarbons by *Bacillus cereus*, although the mechanism(s) involved the expression of specific transmembrane proteins associated with membrane transport and motility [38]. Our results strongly suggest that metal removal mechanism(s) induced by Tween^®^ 80 differ from those induced by the adsorption of ion-exchange compounds on the bacterial surface (see below).

### 3.2. Mechanisms Involved

Tween^®^ 80’s possible reduction of bacterial cell aggregation (facilitating the access of metal ion to a greater cell wall surface area) is unlikely to account for the enhanced removal observed here. Furthermore, a reduction in surface tension and metal solubility does not explain why no Tween^®^ 80-associated effect was seen with Gram-negative and other bacteria. In contrast, Tween^®^ 80 is known to reduce cell membrane permeability in lactobacilli [28]. When used as a growth medium supplement or added immediately prior to exposure to metal ions, Tween^®^ 80 might influence the bacterium’s membrane properties and fatty acid profiles. Although the oleic acid moiety of Tween^®^ 80 can be effectively incorporated into the cell membrane, these levels are marginal. Given that metal removal is enhanced by short bacteria–detergent contact times (as little as 5 min) and low detergent concentrations, de novo expression of surface proteins is unlikely to be involved. It seems more likely that the detergent is responsible for surface changes and membrane disorganization. The fact that shaving bacterial surface proteins enhances Pb(II) removal suggests that the baseline covering of innate proteins (i.e., the dominant S-layer) hinders the uptake of Pb(II) ions into the cytoplasm. Thus, detergents might act by disrupting the membrane and dissociating protein-protein interactions. However, the failure of deoxycholate (a bile salt often used to solubilize membrane proteins) to enhance metal uptake does not fit with this hypothesis. We cannot rule out a contribution of cell surface electronegativity (which increases slightly after “shaving” or after treatment with Tween^®^ 80) to the facilitated entry of Pb(II) located close to the cell wall. However, the zeta potential variations were small and do not fit with the all-or-nothing phenomena reported here. Lastly, microscopy did not reveal obvious surface changes or explain the selective enhancement seen for lead. However, we did not fully elucidate the complete mechanism(s), and further studies are now warranted. Nevertheless, the effect described here should open up some interesting applications.

### 3.3. Possible Applications

Despite the absence of a clear mechanistic explanation, one can imagine several interesting uses for Tween^®^ 80-treated *P. freudenreichii*. Firstly, the selectivity regarding Pb(II) might enable the selective separation of Pb from solutions also containing other metals. It is not appropriate to use Langmuir adsorption models to compare Pb(II) removal in Tween^®^ 80-treated *P. freudenreichii* strains vs. other bacteria because most of the Pb(II) is internalized and not bound to the surface. As suggested for lactobacilli [39], detergent-modified *P. freudenreichii* might be of use in the bacterial remediation of lead from polluted environments, wastewater, and contaminated foods. Furthermore, *P. freudenreichii*’s probiotic-like properties prompt us to recommend it for safe in vivo applications in animals and humans [10,40]. For example, the clay-based intestinal adsorbents used for the symptomatic treatment of diarrhea contain traces of lead and are thus not recommended for children below 2 years of age, pregnant women, and breastfeeding women. In this context, Pb could be detoxified inside the gut by applying a dual anti-inflammatory/anti-infectious bacterium such as *P. freudenreichii* [41].

## 4. Materials and Methods

### 4.1. Chemicals and Reagents

Chemicals and reagents: Tween 20, Tween 80, Nonidet-P40, Triton X-100, the sodium salt deoxycholate, the sodium dodecyl sulfate (SDS), the 3-((3-cholamidopropyl) dimethylammonio)-1-propanesulfonate (CHAPS); osmium tetroxide potassium ferricyanide, uranyl acetate, KH_2_PO_4_, were purchased from Sigma-Aldrich Chemical (Saint-Quentin-Fallavier, France), unless otherwise stated. Ultrapure water corresponds to PURELAB Option-Q from Veolia Water (Antony, France).

### 4.2. Bacterial Strains and Culture Conditions

We studied (i) 21 strains of *P. freudenreichii* from the Centre International de Ressources Microbiennes-Bactéries d’Intérêt Alimentaire collection (CIRM-BIA; STLO, INRAE, Rennes, France), which had previously been characterized (using comparative genomics) for their immunomodulatory potential [42]. Formerly, Pf CIRM BIA-1; Pf CIRM BIA-9; Pf CIRM BIA-118; Pf CIRM BIA-119; Pf CIRM BIA-121; Pf CIRM BIA-122; Pf CIRM BIA-123; Pf CIRM BIA-124; Pf CIRM BIA-125; Pf CIRM BIA-129; Pf CIRM BIA-134; Pf CIRM BIA-135; Pf CIRM BIA-138; Pf CIRM BIA-139; Pf CIRM BIA-456; Pf CIRM BIA-508; Pf CIRM BIA-512; Pf CIRM BIA-513; Pf CIRM BIA-514; Pf CIRM BIA-516; Pf CIRM BIA-527. Strains P.f 125 (ITG P14) and P. f 129 (ITG P20) were provided by the Centre International Interprofessionnel de l’Economie Laitière (CNIEL), (ii) three lactic acid bacteria from the DSM collections, (*Lactobacillus plantarum* DSM 13273, *Lactobacillus diolivorans* DSM 14421 and *Pediococcus acidilactici* DSM 19927) which that have been well characterized in comparative genomics studies [43], (iii) three *Escherichia coli* from the ECOR reference collection, and three *Serratia marcescens* laboratory strains (DB10, Jub9 and NS38) and clinical isolates (nine strains from *Bifidobacterium* species *B. adolescentis* FPL 19157; *B. bifidum* FPL 19147 and FPL 19319; *B. breve* FPL 19317 FPL 19318; *B. lactis* FPL 19315; FPL 19316 and Bb12; *B. longum* FPL 19312; FPL 19313 and Morinaga, and four *Cutibacterium acnes* strains FPL 19161; FPL 19293; FPL 19294; FPL 19295) sourced from either fecal samples of human origin (Faculty of Pharmacy of Lille (FPL) collection, University of Lille [26]) or as re-isolates from commercial probiotic bifidobacteria (*B. lactis* Bb12 and *B. longum* Morinaga). For all strains, the species was confirmed using MALDI-TOF. The Ultraflex III matrix-assisted laser desorption/ionization time-of-flight (MALDI-TOF/TOF) instrument and Flex Analysis software were purchased from Bruker Daltonik GmbH (Bremen, Germany). Each strain was referenced by its internal FPL number.

*P. freudenreichii* strains were grown under microaerophilic conditions and without shaking at 30 °C, in yeast extract lactate (YEL) medium [44] supplemented (or not) with various concentrations of Tween^®^ 80, as indicated. Lactic acid bacteria (*Lactobacillus and Pediococcus*) were cultured without agitation in MRS medium supplemented (or not) with 0.1% Tween^®^ 80. *Cutibacterium acnes* strains were grown in YEL broth at 30 °C or 37 °C, depending on their optimal growth temperature (30 °C for *C. acnes* and 37 °C for *Propionibacterium freundenreichii*). Bifidobacteria were grown under anaerobic conditions (using anaerobic generator packs: GENbaganaer, Biomérieux, France) in MRS medium supplemented with 0.1% (*w*/*v*) L-cysteine hydrochloride and (in some cases) 0.1% Tween^®^ 80. Strains of Enterobacterales (*E. coli* and *S. marcescens*) were grown in Luria Bertani (LB) medium at 37 °C without shaking. The culture time required to reach the stationary phase ranged from overnight to 72 h, depending on the species.

### 4.3. Metal Removal Assays

Eight milliliters of stationary phase *P. freudenreichii* cultures was standardized to an optical density (OD, at 600 nm) of 2.5 and then washed twice in Ringer’s solution. The pellets were suspended with 8 mL of the corresponding ion metal solutions (25 ppm PbCl_2_, CdCl_2;_ AlCl_3_, ZnCl_2_, and CuCl_2_) in Ringer’s solution (pH 7.0) and mixed gently (at 12 rpm) at room temperature for 1 h, using a rotary agitator. Here, we used a 1/4-strength RINGER solution (prepared from standardized commercial tablets—Sigma-Aldrich ref 1.155525.0001, Millipore^®^) whose components are NaCl 2.25 g/L, KCl 0.105 g/L, CaCl_2_ 0.12 g/L, and NaHCO_3_ 0.05 g/L. After centrifugation (12 krpm) and two washes in Ringer’s solution, the pellets were suspended in 500 µL of 70% nitric acid, heated at 98 °C for 15 min, and then diluted in mQ water for the ICP-MS metal assay. Metal concentrations in diluted samples were assayed using inductively coupled plasma mass spectrometry (ICP-MS) on a THERMO ICAP^TM^ Qc system (Thermo Scientific, Courtaboeuf, France).

For each strain, the metal removal ability was quantified as the amount of metal in the pellet (expressed as a percentage of the amount in the initial incubation solution: 100 × [(Initial lead concentration = 25 ppm) − (lead concentration in the pellet)]/(Initial lead concentration = 25 ppm). Most assays were performed in triplicate, with three different cultures per strain.

### 4.4. Electrophoretic Mobility (Zeta Potential) Analysis

Bacteria in a 5 mL stationary-phase culture were harvested by centrifugation (8000× *g*, 10 min, room temperature) and washed twice with PBS (pH 7.0). The cell count in the final suspension was approximately 10^8^ CFU/mL. The pellet was resuspended in 2 mL of a 10 mM KH_2_PO_4_ solution (pH 7.0). Electrophoretic mobility was determined according to Schär-Zammaretti and Ubbink’s well-characterized method (Schär-Zammaretti and UK, 2003), using a ZetaSizer nanoZS (Malvern Instruments, Malvern, UK) and with a glass capillary Zetasizer Nanoseries DTS 1061 (Malvern Instruments, Malvern, UK) as the electrophoretic cell. The electrophoretic mobility value was converted into the zeta potential by applying the Helmholtz–Smoluchowski equation [45]. All experiments were performed as biological and technical triplicates.

### 4.5. Enzymatic Shaving of Surface Proteins

Ten milliliters of a stationary-phase *P. freudenreichii* culture (see above) was harvested by centrifugation (6000× *g*, 10 min, 4 °C) and washed in an equal volume of PBS (pH 8.5) containing 5 mM DTT, prior to resuspension (1:10) in the same buffer. Sequencing-grade modified trypsin (V5111, Promega, Madison, WI, USA) was dissolved in the same buffer (qsp 0.2 g/L) and added to the bacterial suspension. Hence, “shaving” was performed by incubation for 1 h at 37 °C in a 0.5 mL reaction volume containing 5 × 10^9^ bacteria and 4 mg of trypsin, with gentle agitation (180 rpm). The bacteria were recovered by centrifugation (8000× *g*, 10 min, 20 °C) and washed three times in PBS, prior to the metal removal assays.

### 4.6. Electron Microscopy

#### 4.6.1. Scanning Electron Microscopy

Bacteria were fixed with 1% glutaraldehyde in saline, washed, treated with 1% osmium tetroxide in water in the dark for 1 h, and then dehydrated in baths with increasing ethanol concentrations. After two baths in 100% ethanol, the bacteria were washed with hexamethyldisilazane and dried on glass coverslips overnight at room temperature. The coverslips were then mounted on stubs and observed with a secondary electron detector in a Zeiss SEM Merlin Compact VP system (Zeiss, Paris, France) operating at 1 kV.

#### 4.6.2. Transmission Electron Microscopy (TEM)

Bacteria were fixed with 1% glutaraldehyde in saline solution. The samples were divided into two pools. The first pool was contrasted with a mixture of 1% osmium tetroxide and 1.5% potassium ferricyanide, followed by 1% uranyl acetate (all in distilled water at room temperature in the dark, for 1 h). After the samples had been washed, they were dehydrated in baths with increasing ethanol concentrations, treated with epoxy resin, and cured at 60 °C for 24 h. Sections (thickness: 70–80 nm) on formvar-coated grids were observed with a Hitachi H7500 transmission electron microscope (Milexia, Saint-Aubin, France), and images were acquired with an AMT digital camera (Milexia, France). To take account of possible interference by the contrasting metals with the lead accumulation experiments, a second (noncontrasted) pool was dehydrated immediately and then treated with epoxy resin.

### 4.7. Statistical Analysis

Graphs were plotted and statistical tests were performed using GraphPad Prism software (version 6.0, GraphPad Software Inc., San Diego, CA, USA). In all cases, experimental groups were compared with their respective controls in a nonparametric, one-way analysis of variance (a Mann–Whitney U test) or a two-tailed Student’s *t*-test, as appropriate. Quantitative variables were quoted as the mean ± standard deviation (SD). The threshold for statistical significance was set to *p* < 0.05.

## 5. Conclusions

Our results evidence a strong enhancement of lead removal from aqueous solution. This phenomenon was specific for the bacterial species and the metal in solution. Further studies are required to fully characterize the underlying mechanisms and the basis of the observed selectivity of detergents and metals. Bioremediation of Pb by *P. freudenreichii* bacteria treated with low Tween^®^ 80 would be a novel, effective, environmentally friendly, sustainable, and cost-effective method for lead remediation. Further research on the ability of specific microorganisms to remove heavy metals or degrade xenobiotics is also warranted.

## Data Availability

The data presented in this study are available on request from the corresponding author.
